# The Role of Response Times on the Measurement of Mental Ability

**DOI:** 10.3389/fpsyg.2022.892317

**Published:** 2022-06-17

**Authors:** Georgios Sideridis, Maisaa Taleb S. Alahmadi

**Affiliations:** ^1^Boston Children’s Hospital, Harvard Medical School, Boston, MA, United States; ^2^Department of Primary Education, National and Kapodistrian University of Athens, Athens, Greece; ^3^Education and Training Evaluation Commission, Riyadh, Saudi Arabia; ^4^National Center for Assessment, Riyadh, Saudi Arabia

**Keywords:** response time, latent class analysis, item response theory, 2PL, 3PL

## Abstract

The goal of the present study was to evaluate the roles of response times in the achievement of students in the following latent ability domains: (a) verbal, (b) math and spatial reasoning, (c) mental flexibility, and (d) scientific and mechanical reasoning. Participants were 869 students who took on the Multiple Mental Aptitude Scale. A mixture item response model was implemented to evaluate the roles of response times in performance by modeling ability and non-ability classes. Results after applying this model to the data across domains indicated the presence of several behaviors related to rapid responding which were covaried with low achievement likely representing unsuccessful guessing attempts.

## Introduction

Several researchers have provided evidence on the usefulness of incorporating response times into our understanding of achievement processes. Increased response times may be indicative of persistence, enhanced effort and active engagement, adaptive motivation, lucky guessing, and drive when individuals engage in academic tasks and empirical evidence has reported positive links between engagement and achievement (Nagy and Ulitzsch, 2021). On the other hand, brief response times may be indicative of avoidance motivation, lack of effort, the desire to withdraw, or faking, and is oftentimes linked to low achievement (e.g., Holden and Kroner, 1992). Several researchers have advocated that response times are another latent contributor to ability (Danthiir et al., 2005; Molenaar, 2015) and, thus, by incorporating response times researchers can enhance accuracy and precision in the measurement of ability. Based on Molenaar (2015) response times encompass information on the response process as they represent a person’s reaction to a stimulus (beginning or process), strategy use (middle), and decision and reporting (end of response process). The present study follows the lead of previous researchers who incorporated response times in our understanding of skills and competencies in educational measurement.

As Erwin and Wise (2002) pointed out, low effort represents the most important obstacle to accurately estimating a person’s abilities. For this reason, several quantitative models have been put forth to increase accuracy and validity. One is the effort-moderated model (Wise and Demars, 2006) in which rapid response is likely reflective of disengagement and effort withdrawal (see also Rios and Soland, 2020). Other attempts to model response times involve the speed-level model (Semmes et al., 2011), the rapid guessing model (Lu et al., 2020), the discrete-time item response model (Otter et al., 2008), the speed-distance model (Ferrando and Lorenzo-Seva, 2007; Ranger and Kuhn, 2012; Fox and Marianti, 2016), the lognormal Response Time model (RT, van der Linden, 2006; van der Linden, 2007), the 2PL compensatory MIRT model for accuracy (Man et al., 2019), and the visual acuity model (Man and Harring, 2019). The goals of these models are oftentimes to identify the associations that low and fast response times entail.

Of particular challenge is understanding the roles and function of response times as they cross beyond a quantitative proxy of effort. For example, a fast response could indicate lucky guessing (Wise and Kong, 2005; Wise, 2019), or high ability levels for which additional time is not required (e.g., San Martín et al., 2006; Ubulom et al., 2012; Foley, 2016). Thus, the same observation (success on an item) could be linked to both high and low achievers’ behaviors. If rapid guessing is unsuccessful, then a fast response is linked to failure, thus, speed is not always adaptive (Haladyna, 2004). On the other hand, a slow response time may indicate a lack of skill and requisite knowledge so that the correct response is not promptly recognized. Slow response may also reflect engagement for the wrong reasons. For example, Sideridis (2006) found that students were engaged but just wandered around, before handing in their exams so that nobody would blame them that they did not try hard enough. In that study, motivated by obligations (strong oughts) partly explained enhanced levels of engagement with the task, albeit at no benefit. Thus, additional time is not always beneficial although in principle more time may point to deep processing of the material, focus, and highly self-regulated behavior. Thus, again both observations may lead to variable behavioral patterns and achievement outcomes. The picture is further complicated by item content, the item difficulty level in relation to person ability, domain interest, and other personal dispositions (e.g., state anxiety), and even more so when we consider time-limited tests. As mentioned earlier, response times in low-high Stakes, attitudes, and traits assessment plays a salient role but has been ignored in the extant literature. The present study utilized a model proposed recently on accommodating response times in the measurement of skills and competencies. The proposed model is discussed next.

### Jeon and De Boeck Mixture Model for the Measurement of Ability Through Incorporating Response Times

Jeon and De Boeck (2019) recently proposed a mixture model on which response times and response vectors are simultaneously estimated to identify ability and non-ability groups. Thus, each latent class is defined by a latent ability factor using the 2PL model, where the probability of correct response Y_ij_ for person j to item i is estimated as follows:


(1) $$P\left(Y_{i\,j}=1|a_{i},b_{i},\theta_{j},R\right)=\frac{e^{\left(-a_{i}\,\left(\theta_{j}-b_{i}\right)\right)}}{1+e^{\left(-a_{i}\,\left(\theta_{j}-b_{i}\right)\right)}}$$

with the probability of person j who belongs to class R being successful on a binary item i being a function of the discrimination parameter a, item difficulty b, and person estimate on the latent trait theta (θ). The term e reflects the exponent function. Eq. 1 can be applied to any latent ability or non-ability group. Item discrimination and item difficulty parameters are derived from an Item Factor Analysis (IFA) as estimated in Mplus 8.7 (Muthén and Muthén, 1998; Asparouhov and Muthén, 2016):


(2) $$\alpha_{i}=\lambda_{i}\,\sqrt{f_{v\,a\,r}}$$

With λ_*i*_ being the item factor loading and $\sqrt{f_{\text{var}}}$ the latent factor variance estimate. When the factor variance is fixed to unity for identification then the item factor loading equals item discrimination. Item difficulty estimates are based on threshold values and are transformed into item difficulties as follows:


(3) $$\beta_{i}=\frac{\tau_{\iota }}{\lambda_{i}\,\sqrt{f_{v\,a\,r}}}$$

With τ_*i*_ being the threshold estimate from the 2PL model, λ_*i*_ being the item factor loading and $\sqrt{f_{\text{var}}}$ the latent factor variance estimate (see Eq. 2). The “secondary” class Sg, defined as a non-ability class is estimated as follows:


(4) $$P\left(Y_{i\,j}=1|\delta_{i},Sg\right)=\frac{e^{\left(-\delta_{i}\right)}}{1+e^{\left(-\delta_{i}\right)}}$$

With δ_*i*_ being the item intercept of the secondary class group and minus that estimate the respective logit. Thus, the probability of success of person j in class Sg is a function of the item difficulty δ_*i*_ but with ability (theta) not factored in. Subsequently, the probability of being assigned to the secondary, no ability class group is estimated using multinomial regression model as follows:


(5) $$P\,\left(S\,g\right)=\frac{e^{\left(\gamma_{0}+\sum_{\iota }^{\text{I}}\gamma_{\iota }^{*}\,R\,T_{i\,j}\right)}}{\sum_{u=1}^{1+s}e^{\left(\gamma_{0}+\sum_{\iota }^{\text{I}}\gamma_{\iota }^{*}\,R\,T_{i\,j}\right)}}$$

With γ_0_ and γ_1_representing intercept and slopes of response times, respectively. The γ_*i*_ parameter shows whether response times contribute to the formation of the Sg class (predicted membership based on RTs). In other words, spending more or less time is a significant piece of information that helps individuals toward being classified in the Sg class. If the γ_*i*_ coefficients are negative throughout the instrument’s items, which would indicate a fast responder group or a “rapid guessing” group. If the gamma coefficients are positive that would be indicative of spending more time on an item without, however, judging the quality of the time. For example, more time could point to persistence and effort, deep processing of the material but also wandering and being engaged out of obligation. Of note here is that assignment of an individual to the Sg group is based solely on the response time construct, which is why this latent group was termed “non-ability.” As is shown later, at times the non-ability group had higher ability compared to the group defined using the latent ability construct.

The present study attempted to evaluate the roles of response times to the achievement of students in the following latent ability domains: (a) verbal, (b) math and spatial reasoning, (c) mental flexibility, and (d) scientific and mechanical reasoning. Specifically, we attempted to identify individuals who are defined by their response times rather than their ability scores using a mixture Item Response Theory (IRT) modeling approach using Mplus. Thus, evaluating the presence of “rapid guessers” and/or “thoughtful responders” was an objective of the present study.

## Method

### Participants and Procedures

Participants were 869 individuals who were administered the Multiple Mental Aptitude Scales, a standardized instrument usually used as an indicator of giftedness. There were 462 females (53.2%) and 407 males (46.8%). The measure assesses four domains, namely (a) verbal (alpha = 0.59), (b) math and spatial reasoning (alpha = 0.66), (c) mental flexibility (alpha = 0.60), and (d) scientific and mechanical reasoning (alpha = 0.40) each comprising of *n* = 13 items. Sample items are shown in Appendix A. Response times were evaluated using computerized means with time being stored on participants’ workstations.

### Models Tested

Four models were engaged and were contrasted with each other using either information criteria or loglikelihood difference tests. These were: M1 = a one-class model, M2 = a two-class model with one ability class, and one non-ability class with success rates equal to 25% as that would be indicative of successful guessing with polytomous data. The third model, M3 = a two-class model with an ability and a non-ability class, freely estimated as a means of identifying the presence of more than one latent groups, and, last, M4 = a two-class model with the ability class being defined using the ability response vector and the non-ability class defined using the response time estimates, so that an ability and a non-ability response-time group would be identified.

## Results

### Construct Validity of Multiple Mental Aptitude Scale: Prerequisite Analyses

**FIGURE 1:**
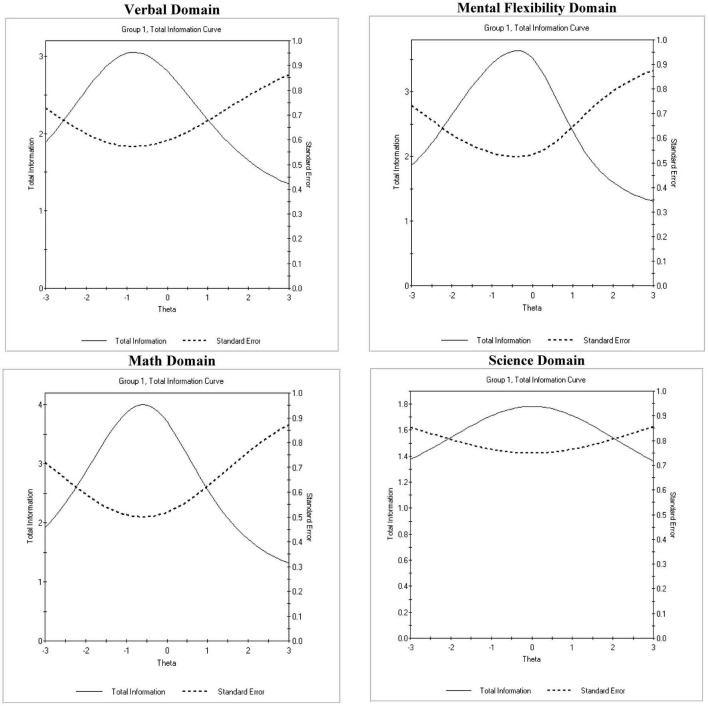
Total information curves (TIF) and conditional standard errors of measurement (CSEM) for each domain of the measure.

A 2PL Item Response (IR) model was fit to the data for each domain separately to verify the unidimensionality and construct validity across domains. Model fit was judged using the Root Mean Square Error of Approximation (RMSEA) for which acceptable values are less than 0.06. Less attention was given to the chi-square statistic given likely high levels of power with a sample of 869 participants, but it is nevertheless presented. Results concerning the verbal domain indicated good model fit using residuals (i.e., RMSEA = 0.04) despite the chi-square test being significant [χ^2^(65) = 154.730, *p* < 0.001]. Total information and conditional standard error of measurement are shown in Figure 1, suggesting that overall, the verbal domain was relatively easy or in other words more accurate for individuals of low ability levels. Results from both the math and science domains indicated that residuals were almost zero (i.e., RMSEA < 0.001) with the omnibus chi-square test also being non-significant pointing to the presence of almost perfect model fit [Math: χ^2^(65) = 65.440, *p* = 0.462: Science: χ^2^(65) = 74.320, *p* = 0.200]. The Math total information peaked around −0.5 logits so, it was relatively easy but covered a wide range of theta. The science domain measure was ideal in that it peaked around zero suggesting that the highest accuracy was at average difficult levels, where most participants are located. Last, the mental flexibility data also showed a good model fit with RMSEA values being 0.04 although with a significant omnibus test [χ^2^(65) = 172.220, *p* < 0.001]. The measure provided its peak information around zero, but accuracy seemed to be higher at lower levels of skill suggesting that the measure was less accurate in capturing theta in the range of +1 and +2 logits.

In order to rule out the hypothesis that high achievement was a function of successful guessing we employed the 3PL model which allows for the estimation of a lower asymptote at the item level. For such a scenario to be operative, significant amounts of non-zero guessing should be present at the item level. Such a fact would be evident using two means (a) significantly improved model fit from estimating the lower asymptote (guessing) compared to the 2PL model, and/or, (b) significant amounts of nonzero guessing at the item level. For the (a) criterion we employed the “fair” Bayesian Information Criterion (BIC) which was consistently lower in the 2PL model compared to the 3PL model across all domains: [Verbal-BIC-2PL = 14379.336; Verbal-BIC-3PL = 14455.352; Math-BIC-2PL = 14053.243; Math-BIC-3PL = 14125.716; Mental Flexibility-BIC-2PL = 13672.166; Mental Flexibility-BIC-3PL = 13695.999; Science-BIC-2PL = 14648.214; Science-BIC-3PL = 14736.170]. For the (b) criterion there were very few items within a domain for which a lower asymptote was justified using a *z*-test (i.e., it was non-zero using a.01 alpha level due to excessive power). There were 1 item in the verbal domain, 2 items in the math domain, 4 in the mental flexibility domain, and 4 in the science domain, out of a total of 52 item tests. Collectively, these findings suggest that the amount of successful guessing was minimal and likely does not inflate the results by representing participants who were successful due to lucky guessing only.

### Measurement of Mental Ability: Model Selection

#### Verbal Domain

Table 1 shows the results from the four tested models with the reference model being a class 1 unrestricted model. M2 added a second non-ability class with the latent factor having zero contribution and the thresholds fixed to what would constitute successful random guessing (i.e., a 25% success rate in multiple-choice items with four options). This model was not nested to the 1-class model but information criteria pointed to a lack of fit for the constrained model as information criteria were saliently larger compared to the unconstrained model (i.e., M1). M3 tested the presence of two latent subgroups (classes), one defined by ability only and one not defined using ability, both freely estimated. This model was superior to the 1-class model using a difference likelihood ratio test [χ^2^(14) = 1218.775, *p* < 0.001] pointing to the presence of two latent subgroups with no apriori known composition. The last model tested the presence of two latent subgroups with one important difference; the non-ability class group was defined using information provided by the response times vectors only (by ignoring ability). M4 was superior to M3 in that the chisquare difference test was equal to 146 chi-square units favoring M4 over M3, which is significant given 12 DF. This finding was further substantiated from estimates of both the BIC and the sample adjusted BIC that were smaller in M4 compared to all other models tested.

**TABLE 1:** Model comparison using difference LRT tests and information criteria across domains of the multiple mental aptitude scales.

Model	LL	N. Par	SCF	M-Comp	−2*LL	DTSC	sLRTS	d.f.	BIC	SABIC
**Verbal Domain**										
M1: 1-Class	−7101.692	26	1.020	–	–	–	–	–	14379.336	14296.766
M2: 2-Classes (1Ab/1nAb) fixed	−7397.826	26	1.463	–	–	–	–	–	14971.603	14889.033
M3: 2-Classes (1Ab/1nAb) free	−7072.979	40	1.137	M3 vs M1	649.694	0.533	1218.775***	14	14416.651	14289.621
M4: 2-Classes with RT	−7004.101	52	1.092	M4 vs M3	137.756	0.940	146.476***	12	14360.104	14194.965
**Math Domain**										
M1: 1-Class	−6938.645	26	0.999	–	–	–	–	–	14053.241	13970.672
M2: 2-Classes (1Ab/1nAb) fixed	−7216.457	26	0.999	–	–	–	–	–	14608.866	14526.296
M3: 2-Classes (1Ab/1nAb) free	−6922.954	40	1.055	M3 vs M1	587.006	1.160	506.189***	14	14116.601	13989.571
M4: 2-Classes with RT	−6880.224	52	1.147	M4 vs M3	85.460	1.452	58.855***	12	14112.350	13947.210
**Mental Flexibility Domain**										
M1: 1-Class	−6748.108	26	1.042	–	–	–	–	–	13672.166	13589.596
M2: 2-Classes (1Ab/1nAb) fixed	−7026.304	26	1.015	–	–	–	–	–	14228.558	14145.988
M3: 2-Classes (1Ab/1nAb) free	−6692.175	40	1.055	M3 vs M1	668.258	1.130	591.543***	14	13655.043	13528.013
M4: 2-Classes with RT	−6616.212	52	1.136	M4 vs M3	151.926	1.404	108.225***	12	13584.326	13419.187
**Science Domain**										
M1: 1-Class	−7236.131	26	1.048	–	–	–	–	–	14648.214	14565.644
M2: 2-Classes (1Ab/1nAb) fixed	−7405.254	26	1.059	–	–	–	–	–	14986.458	14903.888
M3: 2-Classes (1Ab/1nAb) free	−7218.123	40	1.109	M3 vs M1	374.262	1.203	311.211***	14	14706.939	14579.909
M4: 2-Classes with RT	−7170.857	52	1.263	M4 vs M3	94.532	1.777	53.192***	12	14693.615	14528.476

LL, loglikelihood; N.Par, Number of estimated parameters; SCF, scaling correction factor; M-Comp, Model Comparison; −2*LL = −2(LL0−LL1); DTSC = (p0* c0−p1*c1)/(p0−p1); sLRTS = LRTS/dtsc; d.f. = p1−p0.

***p < 0.001; **p < 0.01; *p < 0.05.

#### Math Domain

Similarly in the math domain, there was a preference for the latent class model that incorporated response times (M4) using a loglikelihood difference test [χ^2^(12) = 58.855, *p* < 0.001]. Model 4 was superior to all models except the 1-class model using information criteria, but all inferential models pointed to the superiority of M4.

#### Mental Flexibility Domain

Model 4 was also the preferred choice in the mental flexibility domain. Contrasting models 3 and 4 pointed to the superiority of the latter [χ^2^(12) = 108.225, *p* < 0.001] with both the BIC and SABIC also corroborating the same conclusion.

#### Science Domain

Last, the findings in Science were similar to those in the math domain in that the loglikelihood difference test suggested that M4 was the preferred choice with these data [χ^2^(12) = 53.192, *p* < 0.001], but information criteria favored the 1-class model.

### Evaluating the Roles of Response Times by Ability Domain

Last, the findings in Science were similar to those in the math domain in that the loglikelihood difference test suggested that M4 was the preferred choice with these data [χ^2^(12) = 53.192, *p* < 0.001], but information criteria favored the 1-class model.

#### Verbal Domain

**FIGURE 2:**
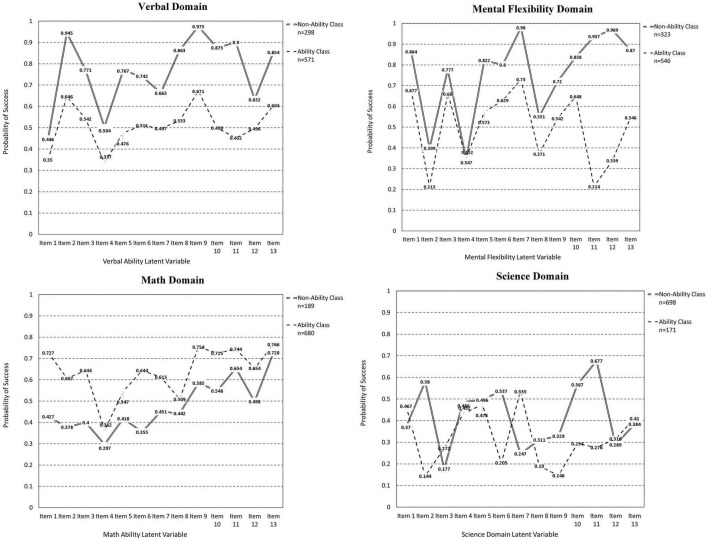
Two class model for the measurement of the 4-latent variable model of ability.

Table 2 displays estimates of slopes and their standard errors, and thresholds for the two classes. Specifically, the ability class had positive slopes verifying the monotonicity assumption in item response models such as the 2PL. When viewing the roles of response times on achievement in the verbal domain several interesting findings emerged. First, the latent class defined by response times (but not ability) had higher achievement levels in the verbal domain compared to the latent class defined by the latent ability factor. This “surprising” finding was also observed in the Jeon and De Boeck (2019) study where a non-ability, the high-achievement group was observed in their knowledge retrieval study. Second, as shown in Table 2, there were significant differences in the response times between the two groups on items 1 and 4–9. As shown in Figure 2, significant between-group differences in verbal ability, given differences in RTs were only observed in items 4 through 9. For items 4 through 7, which were difficult, participants of lower ability spent significantly more time trying to provide correct responses, but the additional time was not beneficial as it was not associated with elevated performance. Performance was significantly lower compared to the higher ability group. That is, although students of lower ability spent more time on items 4–7, their performance remained significantly lower compared to individuals spending less time, who had higher levels of ability. We hypothesize that the additional time spent by low achieving individuals on items 4–7 was not beneficial because it tapped content that was beyond their current skill level. On the other hand, items 8 and 9 were easier overall and participants of low ability spent significantly less time. Thus, in those instances, fast responding was anticipated because shorter times are expected for extreme items (i.e., too easy or too difficult), reflecting an inverted-U shaped relationship between achievement and response times (Akrami et al., 2007; Ranger and Kuhn, 2012). These interpretations, however, should be viewed with caution as they are merely speculative.

**TABLE 2:** Parameter estimates for ability and non-ability classes.

	Defined by ability class				Defined by response times class			
	Slope$\lambda_{\iota }^{R\,g\,1}$		Threshold$\tau_{\iota }^{R\,g\,1}$		Threshold$\delta_{\iota }^{S\,g}$		RT Slope$\gamma_{\iota }^{S\,g}$	
Items	Est.	S.E.	Est.	S.E.	Est.	S.E.	Est.	S.E.
**Verbal domain**								
1	0.190	0.180	0.626	0.099	0.218	0.161	−0.008*	0.002
2	0.399	0.202	−0.624	0.129	−2.840	0.432	−0.021	0.012
3	0.126	0.163	−0.167	0.099	−1.216	0.219	0.000	0.003
4	0.609	0.265	*0.731*	0.124	−*0.016*	0.159	0.012*	0.004
5	0.805	0.307	*0.109*	0.128	−*1.192*	0.210	0.009*	0.004
6	0.715	0.261	−*0.051*	0.126	−*1.056*	0.175	0.027*	0.006
7	1.590	0.683	*0.018*	0.164	−*0.675*	0.169	0.015*	0.005
8	0.173	0.240	−*0.134*	0.126	−*1.837*	0.281	−0.021*	0.008
9	0.338	0.290	−*0.730*	0.158	−*3.653*	0.652	−0.027*	0.009
10	0.456	0.233	0.004	0.131	−1.949	0.280	−0.013	0.008
11	0.282	0.229	0.198	0.128	−2.201	0.415	−0.009	0.007
12	0.118	0.165	0.015	0.101	−0.539	0.166	0.009	0.005
13	0.398	0.191	−0.436	0.119	−1.767	0.228	−0.004	0.005
**Math domain**								
1	0.850	0.148	−*1.125*	0.133	*0.296*	0.332	0.036*	0.014
2	0.800	0.134	−0.496	0.119	0.498	0.267	0.002	0.010
3	1.666	0.290	−0.877	0.158	0.404	0.328	0.019	0.031
4	0.055	0.118	0.567	0.101	0.859	0.342	−0.112*	0.042
5	0.696	0.137	−0.208	0.099	0.333	0.224	0.016	0.032
6	1.424	0.264	−0.814	0.153	0.596	0.335	0.008	0.008
7	0.691	0.145	−*0.508*	0.104	*0.197*	0.306	−0.044*	0.018
8	1.380	0.214	−0.047	0.134	0.233	0.222	−0.016	0.013
9	1.653	0.298	−*1.636*	0.195	−*0.341*	0.355	0.040*	0.018
10	0.898	0.164	−1.132	0.122	−0.194	0.308	0.007	0.034
11	1.031	0.219	−1.287	0.132	−0.637	0.313	−0.020	0.023
12	0.830	0.159	−0.730	0.106	0.008	0.281	0.016	0.026
13	1.336	0.217	−1.571	0.157	−0.983	0.273	−0.008	0.024
**Mental flexibility domain**								
1	1.248	0.275	−*0.958*	0.164	−*1.852*	0.199	−0.013*	0.005
2	0.518	0.205	1.384	0.133	0.408	0.129	0.001	0.004
3	0.921	0.192	−*0.779*	0.133	−*1.246*	0.161	0.017*	0.005
4	0.110	0.147	0.611	0.101	0.630	0.147	−0.004	0.003
5	0.758	0.188	−0.330	0.130	−1.530	0.191	−0.001	0.003
6	0.692	0.176	−0.582	0.115	−1.385	0.168	0.014	0.007
7	1.024	0.256	−1.201	0.182	−3.910	0.628	−0.018	0.014
8	0.479	0.157	0.554	0.114	−0.206	0.135	0.002	0.002
9	0.214	0.136	−*0.169*	0.096	−*0.945*	0.140	0.021*	0.005
10	0.894	0.210	−0.711	0.132	−1.644	0.188	−0.003	0.006
11	−0.120	0.195	*1.305*	0.168	−*2.693*	0.904	−0.020*	0.009
12	0.014	0.230	*0.669*	0.162	−*3.450*	0.755	−0.023*	0.010
13	0.236	0.152	−0.187	0.110	−1.899	0.211	−0.017	0.011
**Science domain**								
1	−0.737	0.777	0.147	0.295	0.534	0.098	−0.016*	0.008
2	−0.963	0.893	*2.083*	0.955	−*0.321*	0.133	0.024*	0.010
3	0.050	0.429	0.986	0.248	1.537	0.125	0.003	0.009
4	−1.311	1.622	0.365	0.440	0.056	0.098	−0.013	0.011
5	0.075	0.381	0.100	0.210	0.016	0.086	−0.005	0.012
6	−0.159	0.557	*1.361*	0.431	−*0.148*	0.095	−0.037*	0.013
7	−0.511	0.448	−0.150	0.316	1.115	0.156	0.014	0.008
8	0.031	0.439	1.449	0.284	0.796	0.102	−0.018*	0.008
9	2.707	2.372	3.399	2.371	0.712	0.128	0.031	0.022
10	1.063	0.771	1.075	0.401	−0.268	0.110	0.002	0.008
11	0.209	0.569	0.974	0.378	−0.738	0.116	−0.007	0.016
12	0.218	0.302	0.783	0.276	0.902	0.106	0.024	0.042
13	−1.071	0.632	0.449	0.362	0.471	0.102	0.037	0.037

Reaction times slopes that were significantly different from zero are shown using a (*). *p < 0.05. Thresholds in italic show differences between latent classes. Shaded cell estimates show items for which there were significant differences in both likelihood of success and response times between latent classes. Positive coefficients in response times $\gamma_{\iota }^{S\,g}$ indicate that the group defined by response times spent significantly more time on a given item. Negative coefficients in $\gamma_{\iota }^{S\,g}$ indicate faster responding in relation to the latent class group defined by ability.

#### Math Domain

In the mathematics domain, the group defined by ability had higher math competency compared to the group defined by reaction time only. Significant differences in response times with accompanying differences in success rates were observed on items 1, 7, and 9. On items 1 and 9, the low achieving group spent significantly more time on these two items of average difficulty level. However, this additional time was not beneficial as it was not associated with high levels of success. This finding suggests that these items engaged more effort as they were not at the extremes of the difficulty distribution but remained outside the skill level of the non-ability latent class group. Item 7 was also associated with lower achievement in the lower ability group, but there was significantly less time spent on that item. For item 7 we speculate that rapid or careless responding may be operative because the additional time emitted by the ability class was associated with elevated success levels by more than 15%.

#### Mental Flexibility Domain

In mental flexibility, the class defined by response times was more successful (high achievers) compared to the class defined by the latent trait. Significant differences in both response times and item-level success rates were observed on items 1, 3, 9, 11, and 12. Among these, items 1, 11, and 12 were relatively easy. In these items, the higher ability group spent significantly less time compared to the low-skill group. Thus, these items had content that one could easily identify with and either knew it or not. Items 3 and 9 were associated with elevated time spent on behalf of the higher-achieving group. Item 3 was relatively easy, and item 9 was of medium difficulty. It appears that spending more time on these two items was beneficial as the additional time seems to be linked to higher levels of success. Again, caution is advised as the content of these items is not available due to item non-disclosure.

#### Science Domain

As shown in Figure 2, response times were not associated with overall differential achievement rates, but differences were evident in two items for which differential response times were linked to differential success rates. These items were items 2 and 6. Both items were extremely difficult for the low achieving group and at medium levels of success for the high ability group. Item 2 was associated with enhanced effort but obviously, the requisite knowledge was not present to move levels of success higher. Item 6 was similarly a very difficult item for the low achieving group where it also spent significantly less time. Thus, it is likely that rapid responding was engaged to avoid exposure to content that lay well beyond the individual’s level of skill. No other differences were evident in the science domain.

## Discussion

The present study represents an effort to identify ability and non-ability groups using mixture modeling for the population of students evaluating their aptitude and considering the presence of giftedness in Saudi Arabia. After examining several academic domains, it was apparent that response times could partly explain the relationship between personal skills and achievement, given item-level properties. This line of research is novel and important as it opens up new avenues toward our understanding of motivated behavior during test-taking.

The most important finding was that incorporating response times was associated with higher accuracy and precision when estimating person abilities compared to ignoring response times. Using inferential statistical criteria, the model (M4) that simultaneously estimated ability and response times was associated with optimal model fit suggesting enhanced accuracy in the estimation of person abilities, compared to ignoring response times. This fact was evidence across all domains.

A second important finding is that information about the response process, along with a person’s success, given the difficulty of an item provides for a more detailed view of an item’s properties and a person’s responses. Overall, it is likely that when items are of medium difficulty, individuals of low-to-medium ability spend more time in an effort to meet the expectations of that item. That was evident on e.g., items 1 and 9 in the quantitative domain, where participants of low ability spent more time trying to solve these items, with the additional effort met with limited success as there were still significant differences between the two groups. Given that specific analytical skills need to be available for a person to be successful in math, it is expected that the additional time would not be particularly beneficial compared to other subjects. For example, on items 4–7 in the verbal domain, the latent class defined by response times spent significantly more time and had significantly higher success levels compared to the ability-defined class suggesting that the additional invested time was beneficial in solving medium difficulty problems. This deep understanding of student behavior and item attributes would have never been possible if response times would be ignored.

A third important finding is that a deeper understanding of individuals’ behavioral response patterns is now possible. By examining response times, strategy use can be understood. For example, additional time may signal the onset of a knowledge retrieval strategy (Jeon and De Boeck, 2019) whereas brief times may suggest “skimming” through keywords, avoidance motivation or learned helplessness (Seligman, 1972) and hopelessness (Abramson et al., 1989). Furthermore, examining response times increases our understanding of engagement and disengagement with the latter being responsible for low proficiency (Wise, 2015) and invalid parameter estimates (Schnipke and Scrams, 1997). Further information could engage person estimates of successful guessing as a means of informing and understanding the time spent with the items. For example, estimating person-based guessing parameters may inform time spent with an item, given an item’s level of difficulty in relation to the person’s ability. In the present study, this latter incorporation was not possible because the 3PL model did not provide improved model fit. In other words, successful guessing was not operative to an extent that would inform estimation of person ability in important ways. A last idea to understanding the causal origins of response times would be examination of aberrant response patterns using person fit statistics (Tatsuoka and Tatsuoka, 1982; Meijer and Sijtsma, 2001). For example, some person fit indices point to the emittance of “creative responding,” “cheating,” or “careless responding” (Karabatsos, 2003). Identifying such individuals and understanding their engagement practices (via estimating response times) may aid our understanding on the relationship between time spent with an item and the likelihood of success.

The present study also presents certain limitations. First, the methodology used herein represents one among several alternatives to modeling response times (e.g., Pokropek, 2016; Man et al., 2018) with their own advantages and disadvantages (Goldhammer et al., 2016). Second, although the examination of response times provides insights on the participant behaviors, those conclusions are correlational in nature. Thus, caution is advised when interpreting the results in that a conclusion that enhanced engagement results in high achievement cannot be supported using the present design. Third, modeling responses and response times is oftentimes cumbersome and requires additional programming as popular software routines are not readily available to model such data. Fourth, despite the good model fit per domain, inspection of the information functions suggested that the science domain was measured with enhanced measurement error compared to the other domains. In the future, it will be important to extend current knowledge through examining individual differences in response times as a function of item content/difficulty through identifying solution strategies employed by individuals. Furthermore, current person-based analyses can enrich our understanding of achievement using covariates and higher-level relationships as proposed by van der Linden (2017).

## Data Availability Statement

The raw data supporting the conclusions of this article will be made available by the authors, without undue reservation.

## Ethics Statement

The studies involving human participants were reviewed and approved by the ETEC Ethics Committee. Written informed consent to participate in this study was provided by the participants’ legal guardian/next of kin.

## Author Contributions

GS conceptualized the study, contributed to the data analyses, and wrote the manuscript. MTA contributed to the data analyses, wrote the quantitative sections, and contributed to the data for the present illustration. Both authors approved the final draft of the manuscript.

## Conflict of Interest

The authors declare that the research was conducted in the absence of any commercial or financial relationships that could be construed as a potential conflict of interest.

## Publisher’s Note

All claims expressed in this article are solely those of the authors and do not necessarily represent those of their affiliated organizations, or those of the publisher, the editors and the reviewers. Any product that may be evaluated in this article, or claim that may be made by its manufacturer, is not guaranteed or endorsed by the publisher.
